# Functional and Structural Brain Alterations in Encephalitis With LGI1 Antibodies

**DOI:** 10.3389/fnins.2020.00304

**Published:** 2020-04-03

**Authors:** Jianping Qiao, Xiuhe Zhao, Shengjun Wang, Anning Li, Zhishun Wang, Chongfeng Cao, Qing Wang

**Affiliations:** ^1^Shandong Province Key Laboratory of Medical Physics and Image Processing Technology, Institute of Data Science and Technology, School of Physics and Electronics, Shandong Normal University, Jinan, China; ^2^Department of Neurology, Qilu Hospital of Shandong University, Jinan, China; ^3^Department of Radiology, Qilu Hospital of Shandong University, Jinan, China; ^4^Department of Psychiatry, Columbia University, New York, NY, United States; ^5^Department of Emergency, Jinan Central Hospital Affiliated to Shandong University, Jinan, China

**Keywords:** anti-LGI1 encephalitis, multimodal MRI, functional connectivity, effective connectivity, white matter microstructure

## Abstract

**Objective:** The purpose of this study was to examine the neural substrates and mechanisms that generate memory deficits, seizures and neuropsychiatric abnormalities in encephalitis with LGI1 antibodies using a data-driven, multimodal magnetic resonance imaging (MRI) approach.

**Methods:** Functional MRI data were acquired from 14 anti-LGI1 encephalitis patients and 14 age and gender matched normal controls. Independent component analysis with hierarchical partner matching (HPM-ICA) was used to assess the whole-brain intrinsic functional connectivity. Granger causality (GC) was applied to investigate the effective connectivity among the brain regions that identified by HPM-ICA. Diffusion tensor imaging (DTI) was utilized to investigate white matter microstructural changes of the patients.

**Results:** Participants with LGI1 antibodies encephalitis presented reduced functional connectivity in the brain areas associated with memory, cognition and motion circuits, while increased functional connectivity in putamen and caudate in comparison to the normal controls. Moreover, the effective connectivity in patients was decreased from the frontal cortex to supplementary motor area. Finally, patients had significant reductions in fractional anisotropy (FA) for the corpus callosum, internal capsule, corona radiata and superior longitudinal fasciculus, accompanied by increases in mean diffusivity (MD) for these regions as compared to controls.

**Conclusion:** Our findings suggest that the neural disorder and behavioral deficits of anti-LGI1 encephalitis may be associated with extensive changes in brain connectivity and microstructure. These pathological alterations affect the basal ganglia and limbic system besides the temporal and frontal lobe.

## Introduction

Encephalitis with leucine-rich, glioma-inactivated 1 (LGI1) antibodies is a disease characterized by progressive memory loss, confusion, sleep disturbances, and problems with behaviors and spatial orientation. The most common clinical symptoms include memory dysfunction, multiform seizures, faciobrachial dystonic seizures (FBDS) and hyponatremia (Irani et al., 2010; van Sonderen et al., 2016; Beimer and Selwa, 2017; Wang et al., 2017). The LGI1 is a protein that binds together two epilepsy-related proteins called ADAM22 (a disintegrin and metalloproteinase 22) and ADAM23 (Kegel et al., 2013). Encephalitis with LGI1 antibodies occurs when antibodies mistakenly attack LGI1 (Gao et al., 2016; van Sonderen et al., 2016).

Prior neuroimaging studies have reported structural and functional neural differences in various brain areas between anti-LGI1 encephalitis patients and normal controls (NC). Several positron emission tomography (PET) studies have reported alterations of basal ganglia hyperintensities in LGI1-autoantibody faciobrachial dystonic seizures (FBDS) (Flanagan et al., 2015; d’Orsi et al., 2018). In addition, structural magnetic resonance imaging (MRI) studies have revealed decreased volumes of the hippocampus, pallidum, nucleus accumbens, brainstem and cerebellum in patients with anti-LGI1 encephalitis (Finke et al., 2017; Miller et al., 2017; Szots et al., 2017). Hippocampal atrophy with further reduced mediodorsal thalamic and posteromedial cortical volumes were reported in the limbic encephalitis associated with antibodies to components of the voltage-gated potassium channel complex (VGKCC-Ab-LE), where LGI1 was the prominent autoantibody (Loane et al., 2019). Another recent study showed that hippocampal dentate gyrus atrophy predicted pattern separation impairment in patients with LGI1 encephalitis (Hanert et al., 2019). Besides, diffusion tensor imaging (DTI) has been extensively applied to unveil white matter abnormalities in diverse neurological diseases (Gatto et al., 2018; Gatto and Weissmann, 2019). The microstructural integrity impairments of the hippocampus (Finke et al., 2017), corona radiata, capsula interna and corpus callosum (Szots et al., 2017) were found in patients with anti-LGI1 encephalitis in the DTI studies. The magnetic resonance spectroscopy (MRS) revealed lower glutamine/glutamate white matter (WM) levels compared with controls (Szots et al., 2017).

Disruptions of large-scale functional networks including default mode network (DMN), sensorimotor, salience and higher visual networks in patients with anti-LGI1 encephalitis have been revealed in the resting-state fMRI study (Heine et al., 2018). Another VGKCC-Ab-LE study in which LGI1 was the main autoantibody demonstrated that patients had reduced posteromedial cortico-hippocampal and interhippocampal functional connectivities which were correlated with memory scores (Loane et al., 2019). A task-based fMRI study in autoimmune encephalitis with FBDS have reported that higher peak FBDS frequency was significantly related to lower hippocampal activity during scene-encoding task (Nantes et al., 2018).

However, the neural mechanisms underlying memory deficits, seizures and neuropsychiatric abnormalities in anti-LGI1 encephalitis remain unclear. Therefore, the aim of this study was to examine the whole-brain functional and structural alterations as well as their correlations with clinical disease severity in encephalitis with LGI1 antibodies using a data-driven, multimodal MRI approach. We applied the independent component analysis with hierarchical partner matching (HPM-ICA) to assess the brain functional connectivity networks. The causal influence between the independent components was estimated by utilizing granger causality method. Diffusion tensor imaging was used to investigate white matter microstructural changes of the patients. The hypothesis was that we would detect brain connectivity and microstructure differences in anti-LGI1 encephalitis within the cortical-subcortical neural systems that support memory, cognition, and motion dysregulation for anti-LGI1 encephalitis.

## Materials and Methods

### Participants

Fourteen participants with anti-LGI1 encephalitis (11 males, 3 females, mean age 55.9 ± 10.6 years) were recruited from the psychological outpatient clinic at the Qilu Hospital of Shandong University (Table 1). Anti-LGI encephalitis was diagnosed by LGI1 antibodies positive in all patients who had serum LGI1 antibodies. The (+) scoring was found in one patient, and (++) scoring was found in thirteen patients. Ten patients were CSF LGI1 antibody positive that showed (+) scoring. Thirteen patients underwent CSF examination with twelve CSF samples showing a normal white cell count. Four patients had increased protein concentrations (Four patients: 0.75, 0.46, 0.53 and 0.5 g/L [normal: <0.45 g/L]). Six patients had increased lactic acid concentrations (Six patients: 2.7, 2.5, 2.3, 2.5, 2.5, and 2.3 mmol/L [normal: 1.2–2.1 mmol/L]). Ten patients had memory impairment. Eleven patients experienced seizure and four patients had mental and behavioral changes. The FBDS occurred in four patients. All anti-LGI1 encephalitis participants had been diagnosed by a licensed neurology pathologist before enrollment. We evaluated symptom severity on the day of MRI scan using the assessment of the modified Rankin Scale (mRS) for all patients. The mental and cognitive functions of patients were assessed by Mini-Mental State Examination (MMSE) and Montreal Cognitive Assessment (MoCA).

**TABLE 1:** Demographic and clinical characteristics of anti-LGI1 encephalitis patients.

Age, mean (SD)	55.9 (10.6) years
Sex	11 male, 3 female
Modified Rankin Scale score^a^, mean (SD)	2.2 (0.8)
Time from symptom onset to diagnosis, mean (SD)	3.4 (2.3) months
Mini-Mental State Examination (MMSE), mean (SD)	22.1(5.9)
Montreal Cognitive Assessment (MoCA), mean (SD)	18.3(5.5)
**Symptom**	
Memory impairment	10/14 (71%)
Seizure	11/14 (79%)
Faciobrachial dystonic seizures	4/14 (29%)
Mental and behavioral abnormalities	4/14 (29%)
**Cerebrospinal fluid**	
Glucose (mmol/L), mean (SD)	4.6 (1.2)
Chlorine (mmol/L), mean (SD)	121 (5.6)
Protein^b^ (g/L), mean (SD)	0.38 (0.17)
Lactic acid^c^ (mmol/L), mean (SD)	2.2 (0.3)
**Antibodies to LGI1**	
Serum (positive)	14/14 (100%)
Cerebrospinal fluid (positive)	10/14 (71%)

*^*a*^The criteria of the modified Rankin Scale (mRS) score can be described as: 0 – No symptoms at all. 1 – No significant disability despite symptoms; able to carry out all usual duties and activities. 2 – Slight disability; unable to carry out all previous activities, but able to look after own affairs without assistance. 3 – Moderate disability; requiring some help, but able to walk without assistance. 4 – Moderately severe disability; unable to walk and attend to bodily needs without assistance. 5 – Severe disability; bedridden, incontinent and requiring constant nursing care and attention. ^*b*^CSF Protein normal values: <0.45 g/L. ^*c*^CSF lactic acid normal values: 1.2–2.1 mmol/L.*

Fourteen group-matched by age and sex normal controls (9 males, 5 females, mean age 55.5 ± 9.3 years) were recruited by public advertisement to take part in the study. All participants were right-handed, native Chinese speakers. The safety screening form and informed consent form were approved by the Institutional Review Board of Qilu Hospital of Shandong University. The written informed consents were obtained from all participants.

### Image Acquisition

Imaging was performed on a Siemens Verio 3.0 Tesla MRI scanner (Siemens, Erlangen, Germany) with a 32-channel head coil at the Qilu Hospital of Shandong University. Participants were instructed to rest with their eyes closed but not to fall asleep during scanning. Foam cushions were used to reduce head movement. We acquired the resting-state functional MRI data using a single-shot gradient-echo echo-planar imaging (EPI) sequence with the following parameters: repetition time (TR) = 2000 ms, echo time (TE) = 30 ms, flip angle = 90°, field of view (FOV) = 24 cm × 24 cm, matrix size = 64 × 64, voxel size = 3.4 × 3.4 × 4.0 mm, slice thickness = 3 mm. Thirty-six axial slices were acquired aligned the AC-PC plane. The acquisition time was about eight minutes, resulting in a total of 240 volumes. The DTI scanning parameters were as follows: 65 diffusion directions in the axial plane, TR = 6400 ms, TE = 98 ms, flip angle = 90°, FOV = 24 cm × 24 cm, matrix size = 128 × 128, voxel size = 2 × 2 × 2 mm^3^, *b*-value = 1000 s/mm^2^, slice thickness = 3 mm, no slice gap.

### The Functional MRI Image Analysis

The resting-state fMRI image analysis consisted of five procedures: preprocessing of functional imaging data, spatial independent component analysis (ICA) of the preprocessed data, identification of reproducible ICA components, statistical comparison of the processed ICA components, granger causality analysis of the ICA components.

The fMRI images were preprocessed using SPM12 (Welcome Department of Imaging Neuroscience, London, United Kingdom) that was run under MATLAB. The slice timing correction was performed to correct phase shifts between slices caused by interleaved scans. Then motion correction was conducted to correct for head movements. After that, all images were normalized to the Montreal Neurological Institute (MNI) coordinate system and smoothed by an isotropic Gaussian kernel of 8 mm full-width at half-maximum.

The spatial independent component analysis with hierarchical partner matching (HPM-ICA) (Wang et al., 2011; Qiao et al., 2015, 2017) was performed on the preprocessed data to explore the functional connectivity networks. In detail, spatial ICA was firstly used to generate N components for each participant in which the number of sets of independent components (ICs) N was determined by information criteria. The minimum description length and Akaike’s information criterion were combined to estimate the lower and upper bounds of the numbers of ICs, defining an interval for the number N of components. Secondly, the hierarchical partner matching was performed to identify independent components that were reproducible in their spatial configuration across all individuals.

Statistical analysis was implemented to detect random effects of group difference in functional connectivity between anti-LGI1 encephalitis patients and normal controls. The z-score maps of the identified reproducible ICs were entered into a second-level factorial analysis, covarying for age and sex. The uncorrected *p*-value of 0.001 with cluster extent threshold of 30 voxels (determined by Monte Carlo simulation) was used for the correction of multiple comparisons. The Pearson’s correlation analysis was also performed in patients to investigate the correlation between the severity (mRS score) and functional connectivity.

Granger causality was carried out to analyze causal influences across the ICs identified by HPM-ICA method. The granger causality indices (GCIs) were computed using the time courses of the identified ICs (Wang et al., 2011; Qiao et al., 2018). The two-sample *t*-tests were finally used to detect group difference in GCIs between patients and normal controls in which age and sex were applied as covariates.

### Diffusion Tensor Imaging Analysis

The DTI images were analyzed using FMRIB Software Library (FSL) software^1^. We first performed eddy-current correction on DTI data for each participant to adjust the distortions and motion artifacts. Then we used the brain extraction tool (BET) (Smith, 2002) to extract brain tissue from the eddy-current-corrected B0 image and generate brain mask. The FMRIB Diffusion Toolbox (FDT) was subsequently used to reconstruct the diffusion tensor and calculate the fractional anisotropy (FA) and mean diffusivity (MD) map for each participant.

The voxel-wise statistical analysis of the DTI images was performed using tract-based spatial statistics (TBSS) (Smith et al., 2006). First, FA maps of all subjects were aligned to the standard MNI152 space through a non-linear registration. Second, the mean FA image was created and skeletonised. A threshold of 0.2 was used to exclude non-skeleton voxels and generate the mean skeleton. The aligned FA and MD map of each subject were then projected onto the skeleton. Finally, permutation-based non-parametric inference with 5,000 permutations was adopted to identify the differences in the FA and MD images between patients and controls. Age and sex were entered as covariates in the statistical analysis. The threshold-free cluster enhancement (TFCE) was used as multiple comparison correction (Smith and Nichols, 2009; Winkler et al., 2016). A family wise error corrected *P* < 0.05 was considered statistically significant.

## Results

### Reproducible Independent Components

**FIGURE 1:**
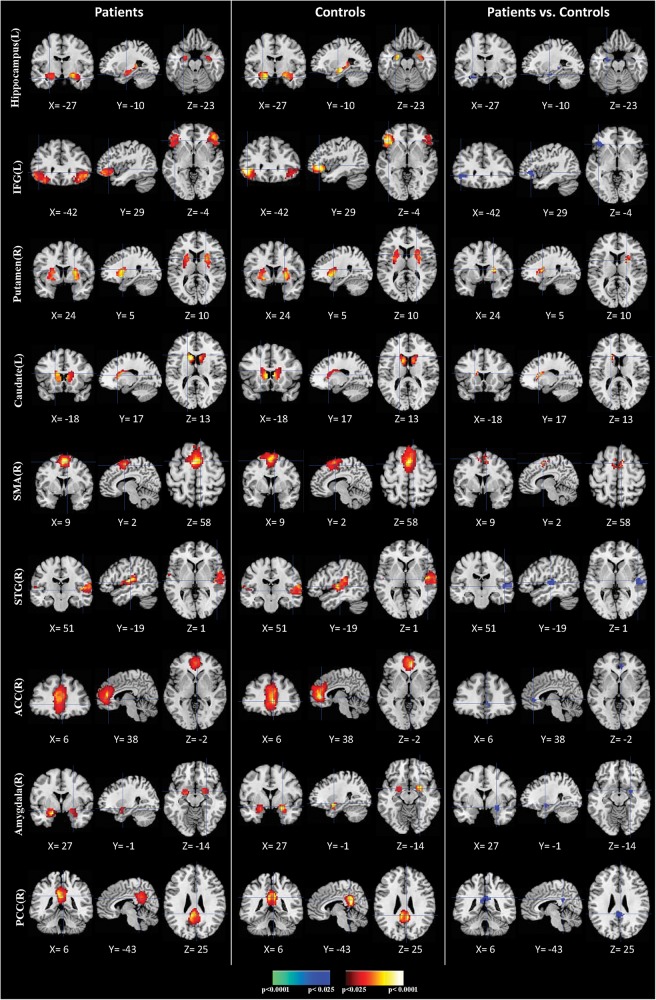
Comparisons of brain connectivity between patients and controls. The first three columns display the connectivity maps detected from the patients group. The second three columns display the connectivity maps detected from the controls group. The last three columns display *t*-contrast maps comparing the group connectivity maps from the patients and controls. IFG, inferior frontal gyrus; SMA, supplementary motor area; STG, superior temporal gyrus; ACC, anterior cingulate cortex; PCC, posterior cingulate cortex.

We identified nine clusters of ICs that were significantly reliable and reproducible in their spatial patterns across anti-LGI1 encephalitis patients and normal controls groups by HPM-ICA method. The general linear model in SPM was applied to conduct a one-sample *t*-test on each of the clusters to generate nine independent component maps that represented statistically significant functional connectivity. The nine ICs of patients and controls were then compared in a second-level analysis. Compared with controls, patients showed significantly reduced connectivity in hippocampus, inferior frontal gyrus (IFG), amygdala, superior temporal gyrus (STG), anterior cingulate cortex (ACC) and posterior cingulate cortex (PCC), but increased connectivity in caudate, putamen and supplementary motor area (SMA) (Table 2 and Figure 1). The higher disease severity (mRS score) correlated with the weaker functional connectivity in the left hippocampus in patients (*r* = 0.76, *p* < 0.01).

**TABLE 2:** Regional locations and significant comparisons of the independent component maps between patients with anti-LGI1 encephalitis and normal controls.

			Peak			*T*
Brain areas	Location		location			statistic
	Side	BA	*x*	*y*	*z*	
**Patients vs. controls (negative)**						
Hippocampus	L	NA	−27	−10	−23	−3.13
Inferior frontal gyrus (IFG)	L	47	−42	29	−4	−3.62
Amygdala	R	NA	27	−1	−14	−3.25
Superior temporal gyrus (STG)	R	22	51	−19	1	−3.42
Anterior cingulate cortex (ACC)	R	32	6	38	−2	−2.71
Posterior cingulate cortex (PCC)	R	23	6	−43	25	−2.59
**Patients vs. controls (positive)**						
Caudate	L	NA	−18	17	13	+ 4.57
Putamen	R	NA	24	5	10	+ 3.13
Supplementary motor area (SMA)	R	6	9	2	58	+ 3.76

*NA, not applicable. All coordinates are in the MNI (Montreal Neurological Institute) ICBM152 space.*

### Granger Causality Interactions

The granger causality index was used to assess the effective connectivity between the regions associated with memory, cognition and motion dysregulation brain networks. The patients had decreased effective connections from the IFG to PCC [(0.15 ± 0.02 versus 0.07 ± 0.01; *p* = 0.01) (mean ± std)], from the IFG to SMA (0.12 ± 0.03 versus 0.07 ± 0.02; *p* = 0.01), while increased effective connectivity from the SMA to caudate (0.16 ± 0.03 versus 0.36 ± 0.09; *p* = 0.007).

### White Matter Microstructure Integrity Alterations

**FIGURE 2:**
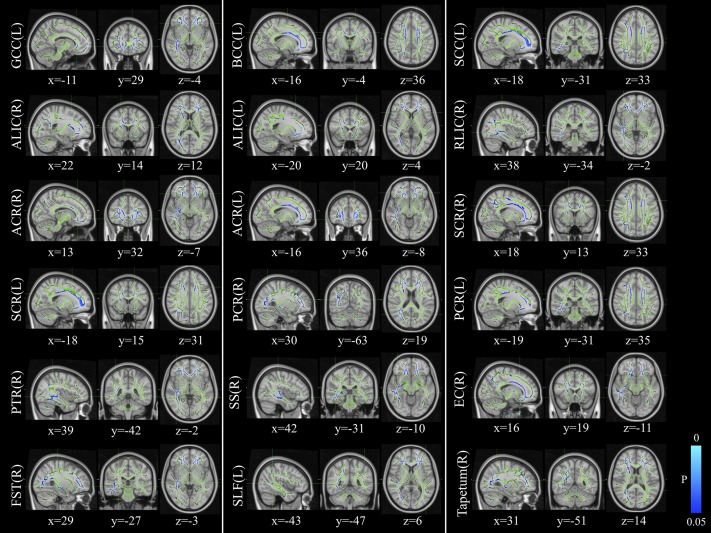
Distribution map of the fractional anisotropy (FA) value that was significantly lower in the patients group compared with the control group based on the TBSS analysis (blue-lightblue). Statistical images (*p*_FWE_ < 0.05 corrected for multiple comparisons) were overlapped onto the mean of the skeleton (green) and the MNI152 template (gray-scale) for visualization. TBSS, tract-based spatial statistics; FST, fornix/stria terminalis; PTR, posterior thalamic radiation; SCR, superior corona radiate; ACR, anterior corona radiate; ALIC, anterior limb of internal capsule; GCC, genu of corpus callosum; SLF, superior longitudinal fasciculus; SS, sagittal stratum; PCR, posterior corona radiate; BCC, body of corpus callosum; EC, external capsule; SCC, splenium of corpus callosum; RLIC, retrolenticular part of internal capsule.

**FIGURE 3:**
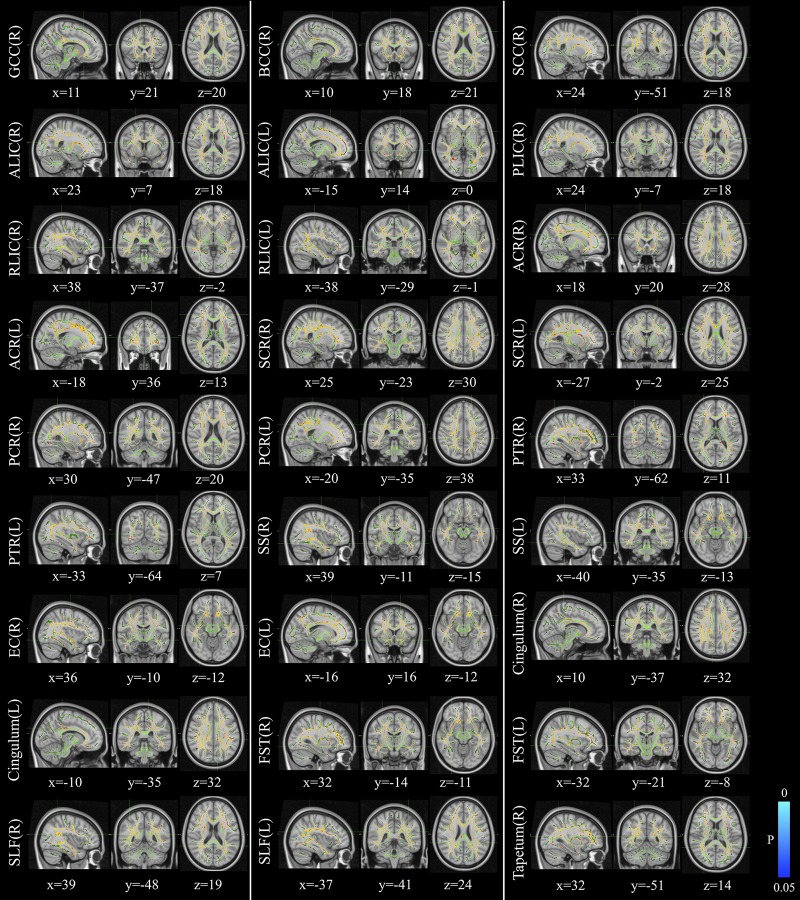
Distribution map of the mean diffusion (MD) value that was significantly higher in the patients group compared with the control group based on the TBSS analysis (red-yellow). Statistical images (*p*_FWE_ < 0.05 corrected for multiple comparisons) were overlapped onto the mean of the skeleton (green) and the MNI152 template (gray-scale) for visualization. TBSS, tract-based spatial statistics; GCC, genu of corpus callosum; BCC, body of corpus callosum; SCC, splenium of corpus callosum; ALIC, anterior limb of internal capsule; PLIC, posterior limb of internal capsule; RLIC, retrolenticular part of internal capsule; ACR, anterior corona radiate; SCR, superior corona radiate; PCR, posterior corona radiate; PTR, posterior thalamic radiation; SS, sagittal stratum; EC, external capsule; FST, fornix/stria terminalis; SLF, superior longitudinal fasciculus.

The voxelwise statistical group comparison between patients and controls showed significantly lower FA in patients for the genu, body, and splenium of corpus callosum, anterior limb, retrolenticular part of internal capsule, external capsule, corona radiata, posterior thalamic radiation, sagittal stratum, fornix/stria terminalis, and superior longitudinal fasciculus (Figure 2 and Table 3), accompanied by increases in MD for these brain regions (Figure 3 and Table 4), as compared to controls. There was a negative association between mRS scores and FA values (*r* = 0.83 *p* < 0.001) and a positive association with MD (*r* = 0.87, *p* < 0.001) in the anterior corona radiate.

**TABLE 3:** Comparisons of FA maps between patients with anti-LGI1 encephalitis and normal controls.

Brain areas	Side	Peak location			*P*-value	Cluster size
		*x*	*y*	*z*		
**Patients vs. controls (negative)**						
Genu of corpus callosum	L	−11	29	−4	0.028	718
Body of corpus callosum	L	−16	−4	36	0.033	742
Splenium of corpus callosum	L	−18	−31	33	0.039	184
Anterior limb of internal capsule	R	22	14	12	0.046	206
	L	−20	20	4	0.040	41
Retrolenticular part of internal capsule	R	38	−34	−2	0.039	128
Anterior corona radiata	R	13	32	−7	0.034	1066
	L	−16	36	−8	0.027	1148
Superior corona radiata	R	18	13	33	0.040	299
	L	−18	15	31	0.033	322
Posterior corona radiata	R	30	−63	19	0.041	80
	L	−19	−31	35	0.039	35
Posterior thalamic radiation	R	39	−42	−2	0.039	653
Sagittal stratum	R	42	−31	−10	0.039	374
External capsule	R	16	19	−11	0.039	137
Fornix/stria terminalis	R	29	−27	−3	0.041	120
Superior longitudinal fasciculus	L	−43	−47	6	0.049	55
Tapetum	R	31	−51	14	0.042	24

**TABLE 4:** Comparisons of MD maps between patients with anti-LGI1 encephalitis and normal controls.

Brain areas	Side	Peak location			*P-*value	Cluster size
		*x*	*y*	*z*		
**Patients vs. controls (positive)**						
Genu of corpus callosum	R	11	21	20	0.008	998
Body of corpus callosum	R	10	18	21	0.008	1537
Splenium of corpus callosum	R	24	−51	18	0.008	859
Anterior limb of internal capsule	R	23	7	18	0.010	387
	L	−15	14	0	0.011	196
Posterior limb of internal capsule	R	24	−7	18	0.010	336
Retrolenticular part of internal capsule	R	38	−37	−2	0.008	358
	L	−38	−29	−1	0.012	231
Anterior corona radiata	R	18	20	28	0.008	1296
	L	−18	36	13	0.009	1374
Superior corona radiata	R	25	−23	30	0.008	1208
	L	−27	−2	25	0.010	999
Posterior corona radiata	R	30	−47	20	0.008	685
	L	−20	−35	38	0.010	600
Posterior thalamic radiation	R	33	−62	11	0.008	655
	L	−33	−64	7	0.010	748
Sagittal stratum	R	39	−11	−15	0.008	464
	L	−40	−35	−13	0.012	335
External capsule	R	36	−10	−12	0.002	635
	L	−16	16	−12	0.010	479
Cingulum (cingulate gyrus)	R	10	−37	32	0.008	75
	L	−10	−35	32	0.049	101
Fornix/stria terminalis	R	32	−14	−11	0.010	70
	L	−32	−21	−8	0.012	112
Superior longitudinal fasciculus	R	39	−48	19	0.008	1196
	L	−37	−41	24	0.010	1104
Tapetum	R	32	−51	14	0.008	36

## Discussion

In this study, we found that patients with LGI1 antibodies encephalitis presented reduced functional connectivities in the hippocampus, IFG, STG, ACC, PCC and amygdala, while increased functional connectivities in putamen, caudate and SMA. Furthermore, the effective connectivity in patients was decreased from the frontal cortex to supplementary motor area. Meanwhile, patients had significant decreased FA and increased MD for the corpus callosum, internal capsule and external capsule, corona radiata, posterior thalamic radiation, sagittal stratum and superior longitudinal fasciculus as compared to controls. The results indicated that abnormal brain connectivity and microstructure in brain areas associated with memory, cognition and motion dysregulation circuits were related to the generic risk of anti-LGI1 encephalitis, which makes it a potential endophenotype for anti-LGI1 encephalitis.

The most commonly brain alteration in anti-LGI1 encephalitis was hippocampal atrophy in the previous MRI studies (Finke et al., 2017; Miller et al., 2017; Szots et al., 2017). Correspondingly, we identified the decreased functional connectivity in hippocampus and its correlation with the disease severity in this study. Moreover, changed functional connectivities were shown in widespread brain regions related to frontal cortex, temporal cortex, motor cortex, basal ganglia and limbic system. These findings were consistent with the previous neuroimaging studies. A resting state fMRI study found disrupted large-scale functional networks including DMNs, sensorimotor, salience and higher visual networks (Heine et al., 2018). The involvement of ACC and frontal lobe in non-paraneoplastic limbic encephalitis has been reported in a recent case study (Ibi et al., 2019). Another PET study showed the hypermetabolism in the anterior cingulate cortex in an anti-NMDAR encephalitis patient (Chanson et al., 2012). The motor cortex has been demonstrated as one of the major signs of LGI1-antibody encephalitis with striatum involvement in parallel (Navarro et al., 2016). In addition, the DTI analysis revealed impaired microstructural integrity in more extensive brain areas compared with the previous surveys (Finke et al., 2017; Szots et al., 2017).

The hippocampus plays important roles in the consolidation of information from short-term memory to long-term memory, and in spatial memory that enables navigation (Eichenbaum, 2017; Lisman et al., 2017; Voss et al., 2017), which is one of the major signs of LGI1-antibody encephalitis (Navarro et al., 2016). The hippocampal atrophy and impaired hippocampal microstructural integrity have been reported in encephalitis with LGI1 antibodies in the previous studies (Finke et al., 2017; Szots et al., 2017). In this study, we identified that patients with anti-LGI1 encephalitis exhibit decreased functional connectivity in hippocampus compared with normal controls, confirming that the alteration of the hippocampus may be related to the development of memory disorders. Furthermore, the correlation of the reduced connectivity in hippocampus with disease severity suggests that the hippocampal damage plays an important role in the symptomatology of anti-LGI1 encephalitis.

We found significantly decreased intrinsic functional connectivity in patients compared to controls in the inferior frontal gyrus and superior temporal gyrus. As one of the major components of executive control functions, the IFG is critical for inhibiting inappropriate motor responses in the framework of top-down control of behavior (Miyake et al., 2000; Swick et al., 2008; Hampshire et al., 2010; Hallam et al., 2018). Evidence has shown that interictal epileptic events were preferentially generated by the temporal or frontal lobes during sleep or drowsiness (Navarro et al., 2016). Therefore, the unsuccessful implementation of inhibitory control over motor responses of the frontal cortex may result in the motor abnormalities such as multiform seizures and FBDS. In addition, the superior temporal gyrus was identified to support auditory short-term memory capacity and speech comprehension ability (Bigler et al., 2007; Leff et al., 2009) as well as social cognition (Guo et al., 2017; Mier et al., 2017). Thus, the functional connectivity reduction in STG may lead to abnormal memory capacity and cognitive impairments in anti-LGI1 encephalitis patients.

We found decreased connectivity in PCC which is the central core of the default mode network (DMN). Increased DMN connectivity has been revealed as a compensatory mechanism for memory impairment induced by hippocampal damage (Heine et al., 2018). The ventral PCC is involved in internally directed cognition such as memory retrieval and planning, while the dorsal PCC has a specific role in modulating the metastability of networks involved in internally directed attention (Leech and Sharp, 2014). The decreased connectivity in PCC may be associated with a reduction in metastability, resulting in an inability to flexibly change between different cognitive states and uncontrolling of attentional focus in patients. In addition, we found reduced functional connectivity in ACC in anti-LGI1 encephalitis patients compared with controls. The ACC is a part of the limbic system and involved in rational cognitive functions, such as reward anticipation, decision-making and impulse control (Agam et al., 2010; Tolomeo et al., 2016). Therefore, the results of our study indicate that the dysregulation of the functional connectivity in ACC might be associated with impaired cognitive control in anti-LGI1 encephalitis.

The anti-LGI1 encephalitis patients exhibited increased connectivity in motor-related brain areas including putamen, caudate and SMA. The striatum which consists of the caudate and the putamen is crucial in the planning and modulation of movement pathways as well as some cognitive processes involving executive function (Zink et al., 2004). The abnormal functional connectivity of striatum may result in the dysfunction of motor generation or control. The decreased effective connectivity from the frontal cortex to supplementary motor area in patients further suggests that the uncontrolled movement in patients may be caused by the disruption of the motor circuits.

Meanwhile, TBSS analysis revealed widespread structural white matter damage in anti-LGI1 encephalitis as assessed using DTI. The corpus callosum (CC) is the largest fiber bundle which transfers motor, sensory, and cognitive information between the brain hemispheres (Bloom and Hynd, 2005; Roland et al., 2017). The genu of CC connects the left and right frontal lobes of the brain. The body and splenium of CC connect the hemispheres of the temporal lobes and the hemispheres of the occipital lobes. We found that anti-LGI1 encephalitis patients had decreased white matter integrity in the genu, body, and splenium of corpus callosum than controls, leading to the lack of movement coordination, low muscle tone, distorted head or facial features, spasms, and seizures. In addition, the internal capsule is a pathway connecting nerves that control the sensation and motor function (Smania et al., 2003). We found the patients exhibited reduced FA in the bilateral anterior limb and right retrolenticular part of internal capsule, which may cause the uncontrolled motor function or the sensation loss in the arm, leg, neck or face of the patients. Moreover, the corona radiata consists of afferent and efferent fibers that connect the cerebral cortex and the brain stem. The decreased white matter integrity of the anterior, superior and posterior of corona radiata in the anti-LGI1 encephalitis patients may affect the sensory input sent from the body to the brain and the messages that are sent from the brain to the body, resulting in the motor and sensory dysfunction of the patients. Furthermore, the superior longitudinal fasciculus is the largest association fiber, which connects the gray matter in frontal, parietal and temporal lobes. It is an important component of the working memory brain network. In this study, the decreased FA in the superior longitudinal fasciculus indicated that the integrity of frontal-parietal network connection was destroyed, which may be the main reason for the significant decline of memory in anti-LGI1 encephalitis patients.

Several limitations should be addressed. First, the sample size in this study was not large, which may decrease the statistical power of our analysis. The future work should be done on more cases to replicate our findings. Second, the patients in the study were all at the acute disease stage, a longitudinal design should be used to address the clinical symptoms and neural alterations with a longer follow-up period such as 3–5 years (Szots et al., 2014). Third, the DTI data processing method using in this study is a mono-exponential approach which has intrinsic limitations to accurately capture intricate WM tracts, particularly around crossing fiber regions, as well as in superficial and deep GM structures. Therefore, other non-Gaussian approaches would potentially be applied on the future clinical investigations such as diffusion kurtosis (Gatto et al., 2019a) and continuous random-walk models (Gatto et al., 2019b) which have been validated in neurological diseases. Finally, it will be important to confirm our results using other techniques, such as EEG-fMRI, which can provide simultaneous cortical and subcortical recording of brain activity with high spatiotemporal resolution.

## Data Availability Statement

The datasets generated for this study are available on request to the corresponding author.

## Ethics Statement

The studies involving human participants were reviewed and approved by Qilu Hospital of Shandong University. The patients/participants provided their written informed consent to participate in this study.

## Author Contributions

JQ, XZ, and SW conceived and designed the experiments. JQ, AL, and QW performed the experiments. JQ and ZW analyzed the data. JQ and ZW contributed reagents, materials, and analysis tools. JQ, AL, CC, and QW wrote the manuscript.

## Conflict of Interest

The authors declare that the research was conducted in the absence of any commercial or financial relationships that could be construed as a potential conflict of interest.

## Footnotes

1 www.fmrib.ox.ac.uk/fsl
